# A Morphological Study of Dynamically Vulcanized Styrene-Ethylene-Butylene-Styrene/Styrene-Butylene-Styrene/MethylVinylSilicon Rubber Thermoplastic Elastomer

**DOI:** 10.3390/polym14091654

**Published:** 2022-04-20

**Authors:** Chunxu Zhao, Xiaohan Chen, Xian Chen

**Affiliations:** Room 602, Yifu Science and Technology Building, Wangjiang Campus, Sichuan University, Chengdu 610065, China; 2020222030015@stu.scu.edu.cn (C.Z.); xc2549@columbia.edu (X.C.)

**Keywords:** thermoplastic silicone rubber, backscattered electrons, compatibility layer, scanning electron microscope, dynamic vulcanization

## Abstract

In this work, we prepared thermoplastic silicone rubber (TPSiV) by dynamically vulcanizing different relative proportions of methyl vinyl silicone rubber (MVSR), styrene ethylene butene styrene block copolymer (SEBS), and styrene butadiene styrene block copolymer (SBS). The compatibility and distribution of the MVSR phase and SEBS/SBS phase were qualitatively characterized by Fourier transform infrared spectroscopy (FTIR) and scanning electron microscopy (SEM) tests on TPSiV. Subsequently, the backscattered electron signal image was analyzed using a colorimeter, and it was found that the size of the interface layer between the MVSR phase and the SEBS-SBS phase could be quantitatively characterized. This method overcomes the defect of the etching method, which cannot quantitatively analyze the size of the compatible layer between the two polymers. The final experiment proved that the two phases in TPSiV exhibited a “sea-island” structure, in which the MVSR phase acted as a dispersed phase in the SEBS-SBS phase. In addition, the addition of the silane coupling agent KH-907 (γ-isocyanatopropyltriethoxysilane) improved the mechanical properties of TPSiV, increasing the tensile strength by about 40% and the elongation at break by 30%. The permanent tensile deformation increase rate was about 15%. Through the quantitative measurement of the compatible layer, it was found that KH-907 could increase the thickness of the interface layer between the MVSR phase and the SEBS-SBS phase by more than 30%, which explained why the silane coupling agent KH-907 improved the mechanical properties of TPSiV at the micro level.

## 1. Introduction

Rubber is a class of polymers that is commonly used in daily life, and vulcanization is a necessary process to impart various properties to rubber [1,2]. Dynamic vulcanization refers to the melt blending of rubber and thermoplastic polymer at a high temperature and then vulcanizing the rubber under the action of a cross-linking agent, thereby obtaining a vulcanized rubber phase with a size in the micron level and uniformly dispersed in the thermoplastic polymer. Compared with other rubber vulcanization methods, dynamic vulcanization has the advantages of greatly improving the performance of blended thermoplastic elastomers, reducing equipment investment, and improving efficiency [3]. Since Gessler first proposed the concept of dynamic vulcanization in 1962, researchers have successively discovered polypropylene (PP)/ethylene propylene diene rubber (EPDM) [4], nitrile rubber (NBR)/polylactic acid (PLA) [5], and other dynamic vulcanization systems.

Silicone rubber is a polymer material with a silicon–oxygen bond as the main chain and phenyl, vinyl, and other groups as side chains and has excellent comprehensive properties [6]. At present, researchers often improve some properties of silicone rubber by blending it with different inorganic substances or polymers. In recent years, the development of special functions, such as high-voltage electrical properties, thermal conductivity, flame retardant properties, and antistatic properties of silicone rubber, has been a trend [7,8]. In addition to silicone rubber, thermoplastic elastomer is widely used because it has the physical and mechanical properties of vulcanized rubber and the processing properties of thermoplastic plastics [9,10]. As one of the most important types of thermoplastic elastomers, SBS is often used for asphalt modification [11]. Since the aging resistance of SBS is not ideal, SEBS obtained by hydrogenating SBS came into being, and it is often used in situations where high aging resistance is required [12]. In this paper, TPSiV prepared by melt blending MVSR and SEBS/SBS is also a material that can combine the biocompatibility of MVSR with the excellent mechanical properties of SEBS/SBS. This TPSiV can be used for medical elastomers and other applications. Before this, the study of this blend system was rarely reported.

Although a variety of materials with excellent properties can be obtained by melting and blending various polymers, because most polymers have poor compatibility with each other, solving the compatibility between polymers and enhancing the interaction between different phase interfaces has become the focus of researchers. Among many solutions, adding a silane coupling agent to the polymer blend system is one of the commonly used methods [13,14]. Therefore, in this paper, we chose the silane coupling agent KH-907 as the compatibilizer of TPSiV.

Scanning electron microscopy (SEM) is one of the most commonly used methods to characterize polymer compatibility and phase interface conditions [15]. The characterization of copolymer compatibility and phase interface by scanning electron microscopy can be roughly divided into two categories. One is to directly observe the surface with obvious phase separation by observing the difference in the phase separation before and after adding the compatibilizer as evidence of the enhanced interaction and improved compatibility of the two-phase interface [16,17,18]. The other is to dissolve the material section in a good solvent of a phase, dissolve and remove the phase, observe the section of the material, and measure the strength of the interaction between the phases by the roughness of the surface of the undissolved phase, to prove that a substance has an obvious effect on the improvement of the compatibility between the two phases [19,20,21]. However, this method is only applicable to the situation where one phase is completely dissolved by the solvent, and the other phase is not dissolved by the solvent at all, which is difficult in some dynamic vulcanization blend systems. In addition, SEM is often used in conjunction with Transmission Electron Microscopy (TEM) and Energy Dispersive X-Ray Spectroscopy (EDX) to characterize more information on the surface of the material [22,23]. However, TEM testing has high requirements for samples, and it is necessary to find suitable dyes.

The SEM test described above usually uses the secondary electron signal [24]. In addition to the secondary electron signal, the backscattered electron signal reflected from the sample surface can also be analyzed to obtain information about the sample surface [25,26]. This characterization method is usually used to observe the changes in alloy phases [27,28,29] and the phase distribution of cement cross-sections [30,31,32]. However, there are few reports on the characterization of dynamic vulcanization blend systems. Therefore, we applied the backscattered electron signal characterization method to the dynamic vulcanization system and quantitatively characterized the phase interface between the dispersed phase and the continuous phase by using the method combined with the colorimeter, which can avoid many shortcomings of the etching method. For example, it is impossible to effectively prove that the etched phase is completely etched, and the unetched phase collapses and easily adheres to the cross-section, which affects the observation and cannot quantitatively characterize the phase interface between the dispersed phase and the continuous phase. At the same time, this method can also observe and analyze the dynamic vulcanization blend system without obvious phase separation.

## 2. Experimental

### 2.1. Materials

To prepare thermoplastic silicone rubber, we used the following materials: methyl vinyl silicone rubber (HCR 9670 UE, Lanxingxinghuo Silicone Co., Ltd., Jiujiang, China), SEBS (YH-602T, Baling Petrochemical Branch of Sinopec Group, Yueyang, China), SBS (D1155JOP, Kraton, Belpre, OH, USA), white oil (250N, Wanghai Petrochemical Co., Ltd., Taizhou, China), silane coupling agent KH-907 (γ-isocyanatopropyl triethoxysilane, Jessica Chemical Co., Ltd., Hangzhou China), platinum catalyst (Daxi Chemical Raw Materials Co., Ltd., Guangzhou, China), and hydrogen-containing silicone oil (hydrogen content 1.6%, Xinglongda New Materials Co., Ltd., Jinan, China).

### 2.2. Sample Preparation

First, the raw materials were weighed according to the weights shown in Table 1. Then, to evenly disperse the white oil 250N in the sample, we first mixed the white oil 250N with SEBS and SBS and stirred for 5 min before letting it stand. After SEBS fully absorbed the white oil, we added the pre-mixed mixture to the torque rheometer (chamber temperature 170 °C, rotation speed 70 rpm). When the torque value was stable, we continued to add the methyl vinyl silicone rubber. We waited for the torque value to stabilize and then added the silane coupling agent, hydrogen-containing silicone oil, and platinum catalyst in sequence to allow for the dynamic vulcanization of the mixture in the cavity. When the torque value rose due to the cross-linking of the mixture in the cavity and then stabilized, the blend was taken out and placed on a flat vulcanizing machine to be pressed for tableting (pressing time was 5 min) to obtain a sample tablet with a thickness of 2 mm.

### 2.3. Scanning Electron Microscope Test

The eight groups of prepared samples were cut into strips with a size of 10 × 2 × 2 mm^3^, and they were placed in liquid nitrogen to freeze for 5 min. Then, the test strips were taken out with tweezers and broken in the middle, and the cross-sections of the samples were treated with gold spraying. Finally, the cross-sections after gold spraying were observed by a scanning electron microscope (Thermo Fisher Scientific, Helios G4 UC, Waltham, MA, USA) to obtain backscattered electron (BSE) images.

### 2.4. Fourier Transform Infrared Spectroscopy Test

The prepared samples were tested with a Fourier transform infrared spectrometer (Bruker Scientific Technology Co., Ltd., Beijing, China). The detection mode was ATR, the resolution was 4 cm^−1^, the test wavenumber range was 400–4000 cm^−1^, and the number of scans was 16.

### 2.5. Etching Method to Test the Cross-Sectional Morphology of the Samples

The eight groups of prepared samples were cut into strips with a size of 10 × 2 × 2 mm^3^, and they were placed in liquid nitrogen to freeze for 5 min. Then, the test strips were taken out with tweezers and broken in the middle. The broken samples were completely immersed in chloroform for 72 h (the samples were taken out and replaced with a new chloroform solvent every 24 h to completely dissolve the etched MVSR phase). After 72 h, the samples were taken out from the chloroform solvent and placed in a fume hood for 12 h to allow the solvent on the surface of the samples to evaporate completely. Finally, the cross-sections were sprayed with gold, and we observed them with a scanning electron microscope (SEM) to obtain the secondary electron images.

### 2.6. Compatibility Layer Quantitative Characterization Test

**Figure 1:**
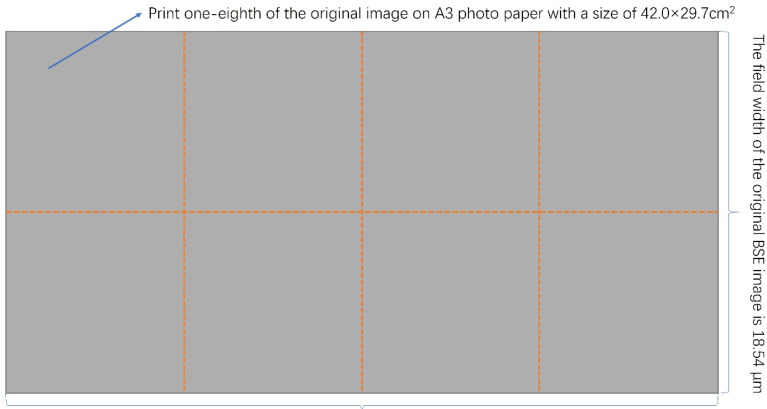
Printing method of BSE diagrams.

**Figure 2:**
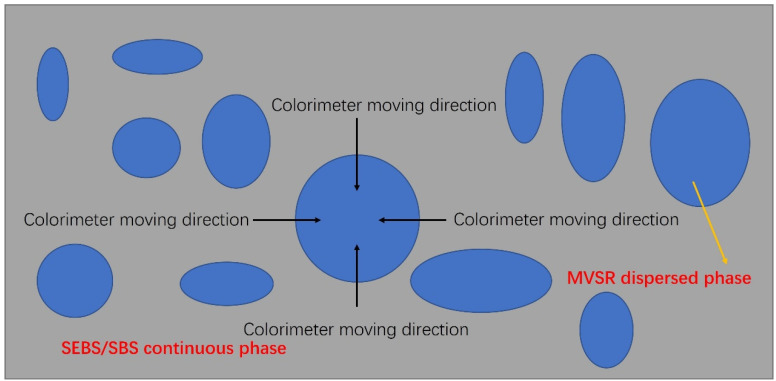
The test method for the colorimeter to test the size of the compatibility layer in TPSiV.

The 1000-times backscattered electron images obtained by the scanning electron microscope test were cut by one-eighth according to the method shown in Figure 1 (image pixel: 3840 × 2160 dpi), and then the obtained images were printed on photo paper by a high-definition printer. The pixel dimensions of the printer were 1200 × 1200 dpi, and the size of the photo paper was 420 × 297 mm. Finally, according to the method shown in Figure 2, the light passage of the colorimeter was oriented and shifted quantitatively to determine the size of the compatibility layer between the MVSR phase and the SEBS/SBS phase.

### 2.7. Mechanical Property Test

According to GB/T 528-2009 standards, five dumbbell-shaped samples with a gauge length of 20 mm, a width of 4 mm, and a thickness of 2 mm were cut from the eight groups of samples prepared in Section 2.2. The tensile strength and elongation at the break of the samples were tested by a tensile testing machine (AGS-J, Shimadzu Corporation, Kyoto, Japan), and the tensile rate was 200 mm min^−1^. Then, the samples were pulled off and left to stand for five minutes, and the permanent tensile deformation was tested after the standing was completed. Finally, the arithmetic means of the test results of the five dumbbell-shaped specimens were taken for each mechanical property test result.

## 3. Results and Discussions

**Figure 3:**
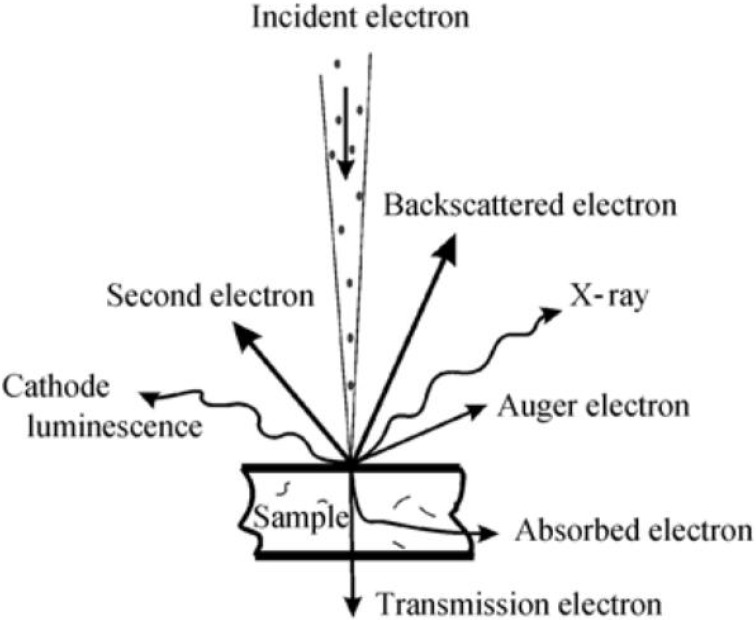
Signals excited when the incident electron interacted with the sample.

The scanning electron microscope (SEM) is a common characterization method to observe the microscopic information of polymer materials. By analyzing the electron signals excited by the interaction of incident electrons with the sample, information about the surface of the sample can be obtained. The excited signal when the incident electron interacts with the sample is shown in Figure 3 [24]. In this work, the condition of the sample surface is mainly characterized by analyzing the secondary electron signal and the backscattered electron signal.

### 3.1. Analysis of Secondary Electron Signal Results of TPSiV Made of MVSR and SEBS-SBS with Different Relative Contents

**Figure 4:**
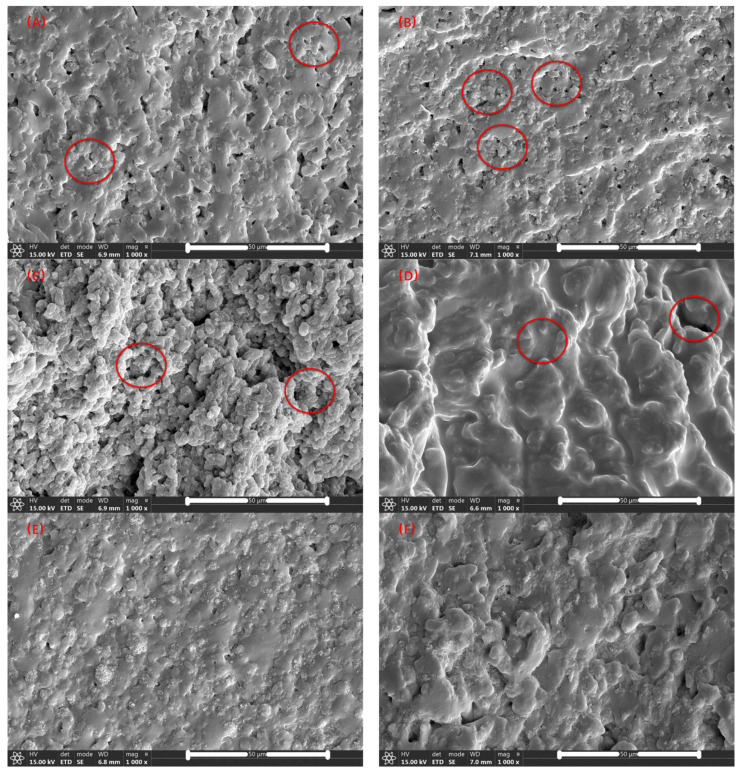

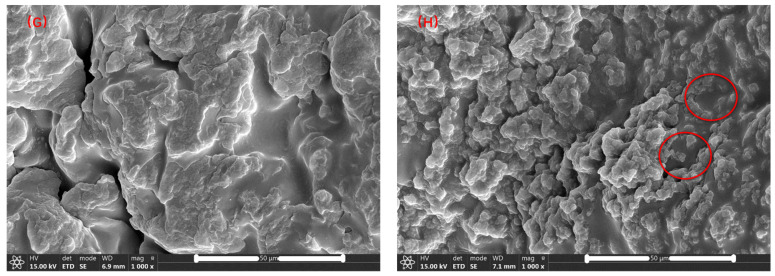
SEM diagrams obtained by etching method (**A**–**H**) correspond to samples 1–8, respectively.

Figure 4A–H are the SEM images of samples 1–8 after being etched by chloroform, during which the MVSR phase was dissolved by the solvent. As the content of silicone rubber increases, the diameter of the pores left by the etching and dissolution of the silicone rubber gradually increases. By observing the marked parts in Figure 4A–D, we can find that with the increase of the content of silicone rubber, the diameter of the pores left by the etching and the dissolution of the silicone rubber gradually increases. However, by comparing the secondary electron signal results of the silane coupling agent KH-907 (for example, Figure 4A,E), we found that the silane coupling agent KH-907 could not distinguish the MVSR and SEBS-SBS interface. Therefore, it cannot explain the improvement of mechanical properties after adding the silane coupling agent KH-907. Except for Figure 4A,B, it is difficult to distinguish the dispersed phase from the continuous phase. Moreover, we observed the marked parts in Figure 4D,H and found that the unetched phase was attached to the cross-section. Since the hardness of the SEBS-SBS phase is relatively small when the relative content of silicone rubber increases, the etching degree of the etched phase also increases, and the remaining SEBS-SBS phase will easily adhere to the cross-section and affect the observation (for example, Figure 4G,H).

### 3.2. Analysis of the Backscattered Electron Signal of TPSiV Made of MVSR and SEBS-SBS with Different Relative Contents

Among many electronic signals, backscattered electrons are the part of primary electrons reflected from the surface of the sample after the elastic and inelastic scattering of the incident electrons from colliding with the atoms on the surface of the sample. Elastically backscattered electrons are incident electrons reflected from the nucleus of the sample after a single or few large-angle elastic scatterings, and their energy does not change. We usually put reflected electrons with slightly varying energies in this category as well. Inelastic backscattered electrons are those incident electrons that are eventually reflected from the sample surface after tens or hundreds of inelastic collisions. Backscattered electrons are reflected in an irregular direction. However, their number is related to the angle of incidence and the average atomic number *Z* of the sample. The larger the value of *Z*, the stronger the signal strength of the backscattered electrons. Experiments show that when the energy of incident electrons is 10–40 keV, the backscattering coefficient *η* of the sample increases with the increase of element atomic number *Z*. The relationship between the backscattering coefficient *η* and the backscattered electron signal intensity *i_b_* and the incident electron intensity *i_p_* is as follows:

(1) $$\eta =\frac{i_{b}}{i_{p}}$$

When the atomic number is greater than 10, the backscattering coefficient *η* has the following quantitative relationship with the atomic number *Z*:

(2) $$\eta =\frac{\ln Z\left(\bar{Z}\right)}{6}-\frac{1}{4}$$

**Figure 5:**
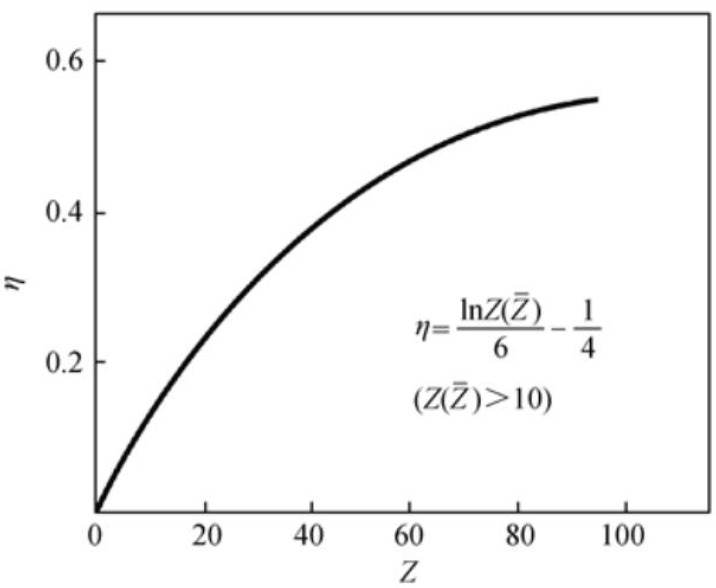
Relationship between atomic number *Z* and backscattering coefficient *η*.

Figure 5 shows the relationship between the atomic number *Z* and the backscattering coefficient *η*. From Figure 5, we can see that for elements with *Z* < 20, *η* increases rapidly and linearly with the increase in *Z*. Therefore, the MVSR phase (the main constituent element is silicon) and the SEBS-SBS phase (the main constituent element is carbon) in TPSiV can be distinguished in the backscattering signal diagram, and the difference in the backscattering coefficient *η* of the two phases is about 0.08 [25].

**Figure 6:**
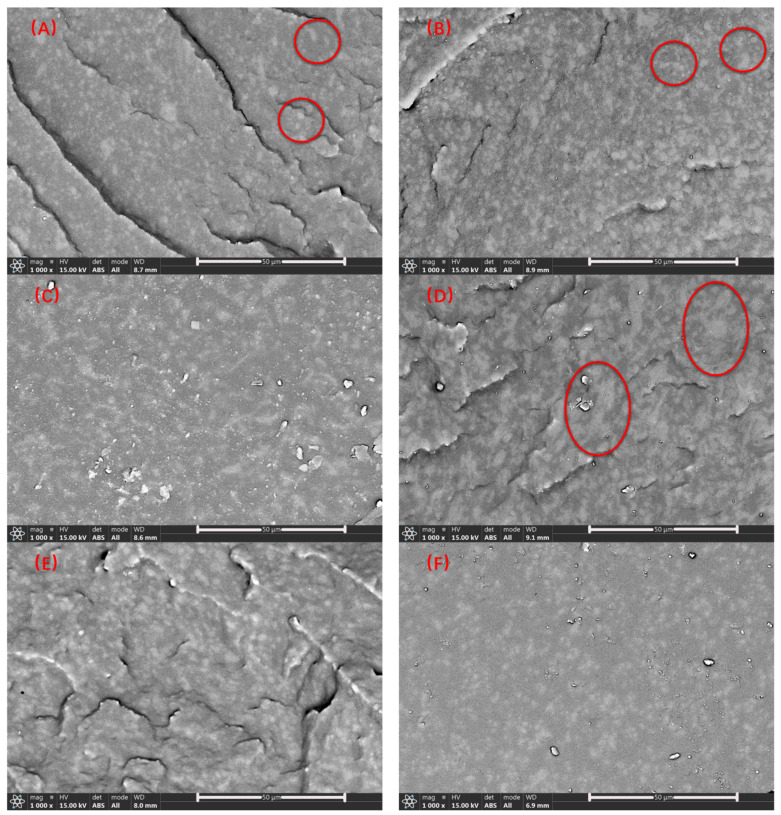

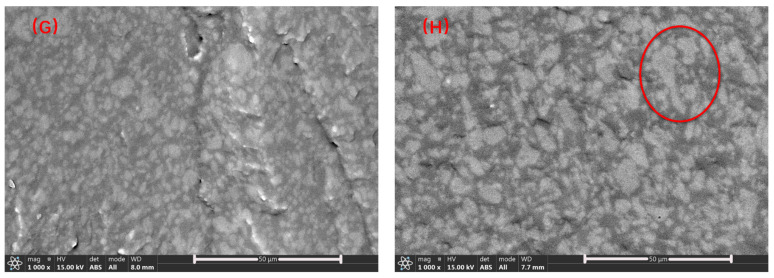
BSE diagrams of TPSiV, (**A**–**H**) correspond to samples 1–8, respectively.

**Figure 7:**
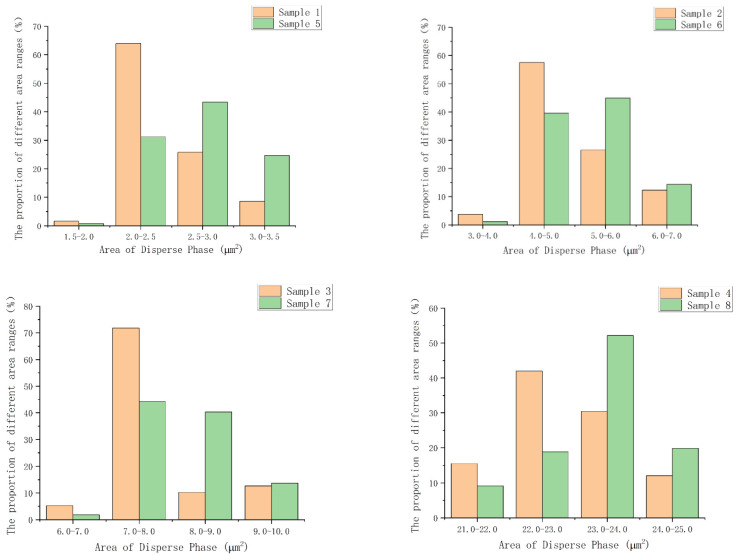
Bar diagrams of the size distribution of the dispersed phase in the backscattered signal diagrams.

The larger the atomic number *Z* is, the stronger the backscattered signal is. Therefore, in Figure 4, the brighter area is the MVSR phase, and the darker area is the SEBS-SBS phase. From Figure 6, we can observe that the atomic number contrast of the two phases in TPSiV achieves the effect of distinguishing them. According to the marks in Figure 6A,B, we also found that when the silicone rubber content was low, a distinct “sea-island” structure was formed between the dispersed phase and the continuous phase. However, as the silicone rubber content increases, especially when the amount of silicone rubber reaches 70 phr, through the observation of the marks in Figure 6D,H, there is a tendency for a co-continuous phase in some regions, and it is difficult to distinguish between the continuous phase and the dispersed phase, which may affect some properties of the material. Through the image analysis system built into the software used when taking the SEM image, we obtained the size distribution of the dispersed phase, as shown in Figure 7. By comparing the dispersed phase size distribution of samples with the same content of silicone rubber and different content of silane coupling agent KH-907 (for example, samples 1 and 5), we found that adding silane coupling agent KH-907 would make the area of the dispersed phase smaller. We think that it may be that the promotion of interfacial interaction by silane coupling agent KH-907 improves the dispersion of the dispersed phase, but this needs to be further verified by other quantitative characterization methods.

### 3.3. Characterization of the Compatibility between MVSR and SEBS-SBS by Fourier Transform Infrared Spectroscopy

Fourier transform infrared spectroscopy is one of the common methods used to qualitatively characterize the compatibility of two polymers after blending. According to the information provided in Table 2, for MVSR, we usually take 1260 and 1010 cm^−1^ as characteristic peaks.

**Figure 8:**
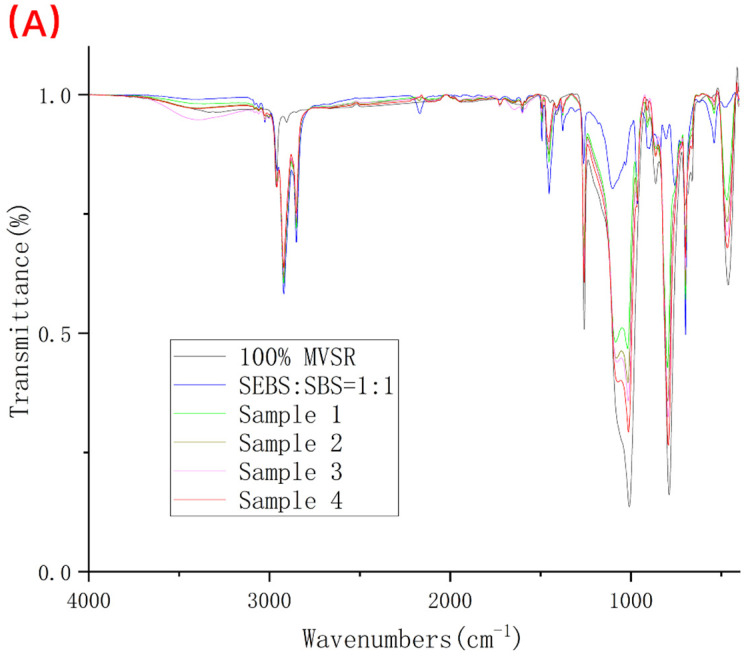

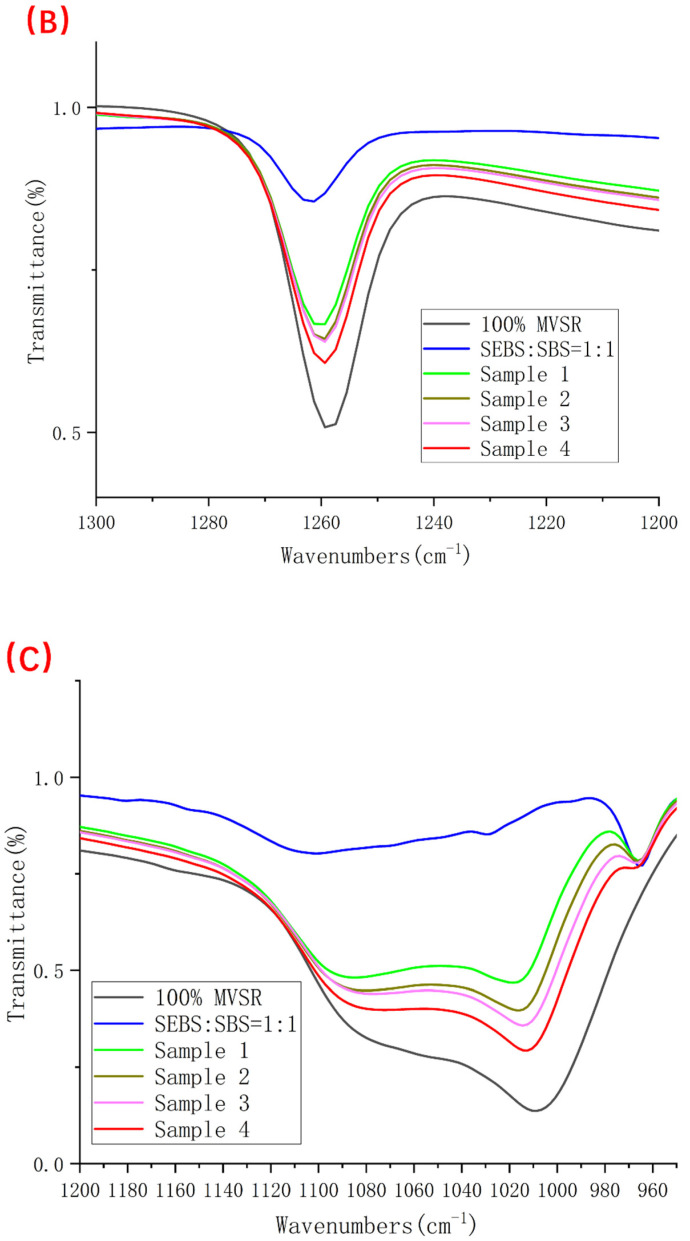
FI-IR diagrams of 100% MVSR, 50% SEBS + 50% SBS and samples 1–4. (**A**) Complete spectrum; (**B**) Enlarged detail near wavenumber 1260 cm^−1^; (**C**) Enlarged detail near wavenumber 1010 cm^−1^.

**Figure 9:**
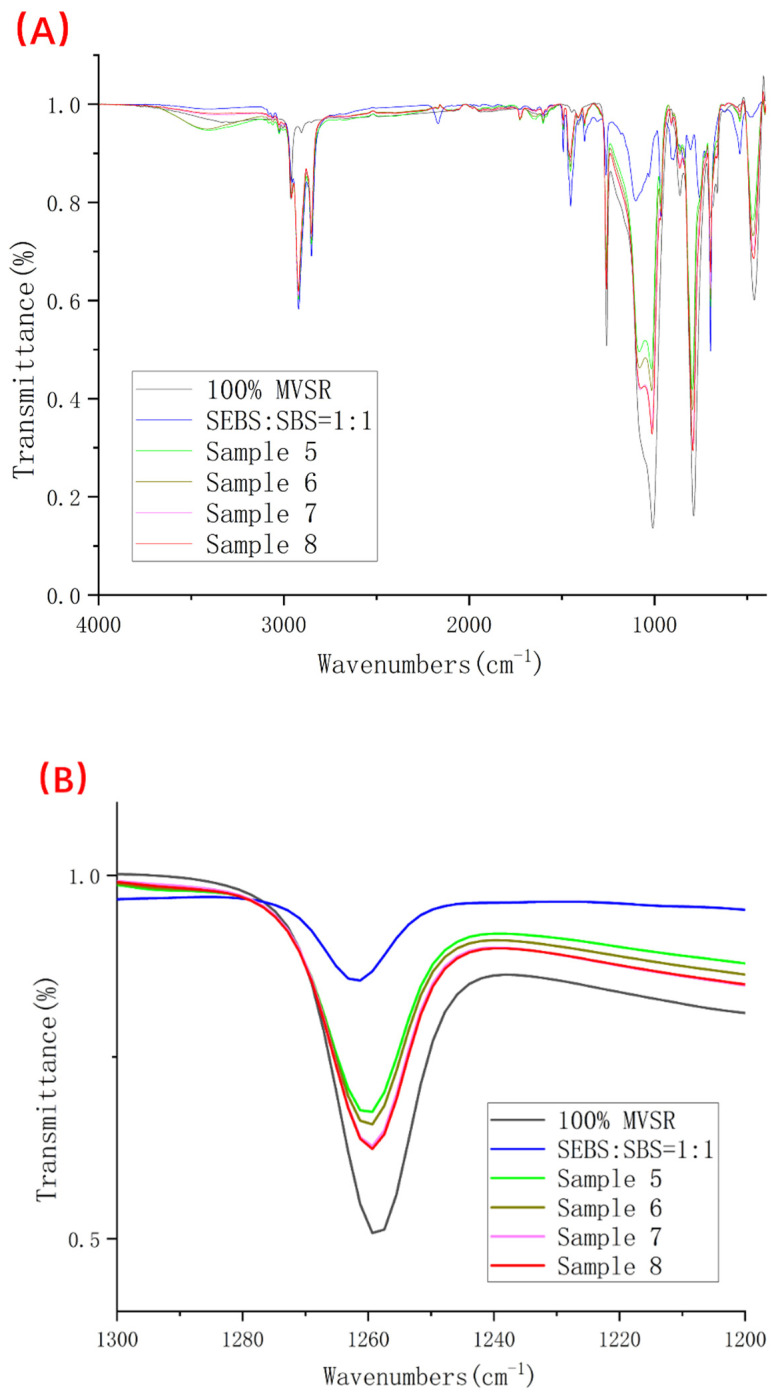

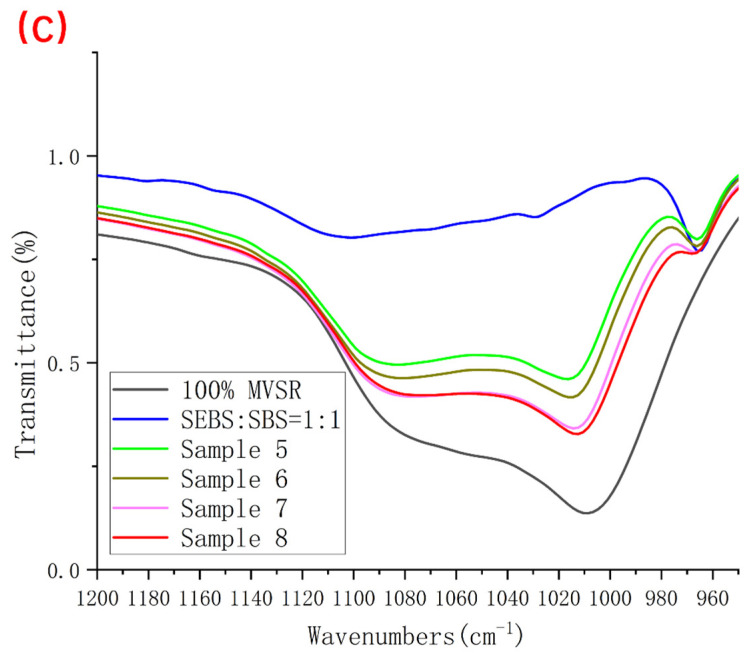
FI-IR diagrams of 100% MVSR, 50% SEBS + 50% SBS and samples 5–8. (**A**) Complete spectrum; (**B**) Enlarged detail near wavenumber 1260 cm^−1^; (**C**) Enlarged detail near wavenumber 1010 cm^−1^.

According to Figure 8 and Figure 9 and Table 3, we found that no matter whether the silane coupling agent KH-907 was added or not, it can bring about the shift in characteristic peaks, and with the decrease in the relative content of silicone rubber, the wavenumber shift is more obvious. However, after adding silane coupling agent KH-907, the value of the wavenumber shift is larger. For example, comparing pure MVSR with samples 1 and 5, the stretching vibration peak of Si(CH_3_)_2_ in sample 1 is shifted by eight wavenumbers compared to the same characteristic peak of pure MVSR, while the stretching vibration peak of Si(CH_3_)_2_ in sample 5 is shifted by six wavenumbers compared to the same characteristic peak of pure MVSR.

Usually, we use the compatibility results obtained by infrared spectroscopy as the basis for qualitative analysis and do not use the absolute magnitude of the wavenumber offset as the basis for the quantitative comparison of the compatibility of the two phases. The infrared spectrum results in this experiment can only show that the blend system of MVSR and SEBS/SBS has certain compatibilities, and we cannot further draw a quantitative conclusion regarding the compatibility of MVSR and SEBS/SBS.

### 3.4. Analysis of the Results of the Quantitative Characterization Test of the Compatibility Layer

We usually use three parameters, *L*, *A* and *B*, to represent the chromaticity value of the color of the object. The size of these three values represents the color space coordinates of a certain color, and every color has a unique color space coordinate value. Among them, *L* stands for lightness and darkness, black and white; *A* stands for red and green; and *B* stands for yellow and blue. Since the BSE image obtained by scanning electron microscopy is black and white—that is, the values of *A* and *B* are both 0—in the experiment, we only measure the chromaticity value of the color of a certain point in the BSE image by the *L* value.

We printed the 1000 x BSE diagrams on A3 size (420 × 297 mm) photo paper with a high-definition printer, as shown in Figure 1, and moved the colorimeter, as shown in Figure 2. In each direction, we recorded the *L* value every time the colorimeter moved by 1 mm and took the arithmetic average of the thickness of the compatible layer obtained from each direction as the final experimental result.

The field of view of the BSE diagram is not related to the size of the photo paper, so we can first find the field of view of the BSE diagram. According to the size of the scale bar in the BSE map and the measurement of the vernier caliper, we can calculate that the field of view of the BSE diagram was 27.19 × 18.54 μm. Next, as shown in Figure 1, we divided the BSE diagram into eight parts, randomly selected one of them, and printed it out on photo paper. According to the segmentation method in Figure 1, we can calculate that the field of view of the divided diagrams was 6.80 × 9.27 μm. We converted this field of view with the A3 size photo paper (420 × 297 mm), and we found that 1 mm length of photo paper represents 0.0162 μm of the diagram length and 1 mm width of photo paper represents 0.031 μm of the diagram width.

Before the start of the experiment, we tested the *L* value of the MVSR and SEBS/SBS phases. We found that the *L* value of the MVSR phase was between 70 and 72, and the *L* value of the SEBS/SBS phase was between 47 and 50. Therefore, we inferred that the distance with the *L* value of 50–70 can be regarded as the thickness of the compatibility layer between the MVSR phase and the SEBS/SBS phase.

According to the data in Table 4, with the increase in the relative content of MVSR, the thickness of the compatibility layer between the MVSR and the SEBS/SBS phases gradually decreased. Since the thickness of the compatibility layer can be used as a measure of the degree of interaction between the MVSR and the SEBS/SBS phases, combined with the statistical data in Figure 7, we believe that the dispersed phase size of TPSiV with a high proportion of MVSR was larger, and the “sea-island” structure tended to disappear because the force of the two phases was reduced. By comparing the thicknesses of the compatibility layers of several groups with the same MVSR content, with the variable only being the content of silane coupling agent KH-907 (for example, samples 1 and 5), we found that the thickness of the compatibility layer significantly increased after adding silane coupling agent KH-907, which shows that the silane coupling agent can promote the interaction between the MVSR and the SEBS/SBS phases. This can serve as a microscopic explanation for the improved macroscopic properties after adding the silane coupling agent KH-907. It can be seen that this characterization method overcomes the disadvantage that the etching method and infrared spectroscopy cannot quantitatively characterize the thickness of the compatibility layer and also avoids the defect that when the MVSR content is high, and the etching method is used, the non-etched phase collapses and then adheres to the section.

### 3.5. Analysis of the Experimental Results of the Mechanical Property Test

Table 5 shows the test results of the mechanical properties of samples 1–8. From this, we can easily find that with the increase in the silicone rubber content, the mechanical properties of TPSiV are significantly reduced. It is more obvious when it increases to 70%. This is because the mechanical properties of silicone rubber itself are poor and far inferior to SEBS and SBS. With the obvious increase in the content of silicone rubber, the material itself gradually showed properties closer to that of silicone rubber. In addition, according to Figure 6D,H, the partial disappearance of the “sea-island” structure in TPSiV is also one of the reasons for the obvious deterioration of the mechanical properties of the sample with a silicone rubber content of 70 phr.

For a material, tensile strength, elongation at break and tensile set are the three most critical mechanical properties. Using the data shown in Table 5, we can analyze the performance change after adding the silane coupling agent KH-907. We can calculate the ratio of improvement in tensile strength and elongation at break by:

(3) $$T=\frac{W_{2}-W_{1}}{W_{1}}$$

where *T* is the improvement ratio of tensile strength and elongation at break, *W*_1_ is the tensile strength and elongation at break before adding silane coupling agent KH-907, and *W*_2_ is the tensile strength and elongation at break after adding silane coupling agent KH-907.

Since the smaller the value of the tensile set, the better the performance of the material, we use the following formula to calculate the improvement ratio of the tensile set:

(4) $$R=\frac{M_{1}-M_{2}}{M_{1}}$$

where *R* is the improvement ratio of the tensile set, *M*_1_ is the tensile set before adding silane coupling agent KH-907, and *M*_2_ is the tensile strength and elongation at break after adding silane coupling agent KH-907.

By substituting the data of Table 5 into Equations (3) and (4), we obtain Table 6. According to Table 6, we found that the addition of silane coupling agent KH-907 significantly improved the mechanical strength of TPSiV; the tensile strength is increased by about 40%, the elongation at break is increased by about 30%, and the tensile set is increased by about 15%. Since we have quantitatively characterized the thickness of the compatibility layer above, we believe that the reason for the improvement of mechanical properties comes from the increase in the thickness of the compatibility layer. When the material is stretched, the interaction between the MVSR phase and SEBS/SBS phase will be closer. This closer interaction gives the material better mechanical properties and resilience.

## 4. Conclusions

We prepared a thermoplastic silicone rubber from methyl vinyl silicone rubber, SEBS and SBS using a torque rheometer. By taking the backscattered electron diagram of the material, we found that the phase distribution of the material could be characterized and analyzed using this method. The MVSR phase is the dispersed phase, the SEBS/SBS phase is the continuous phase, and the two have a “sea-island” structure. When the MVSR content reaches 70 phr, the “sea-island” structure tends to disappear. After adding silane coupling agent KH-907, the size of the MVSR phase will decrease. According to the results obtained by infrared spectroscopy, we found that there is a certain degree of compatibility between the MVSR phase and the SEBS/SBS phase, but this can only be used as a basis for qualitative analysis. By using a colorimeter to analyze the printed backscattered electron diagrams, we found that the size of the compatibility layer between the two phases can be quantitatively characterized. Moreover, the silane coupling agent KH-907 can increase the thickness of the compatibility layer between the two phases. In the subsequent mechanical property test, we also found that silane coupling agent KH-907 improved the mechanical properties of TPSiV, in which the increase rate of tensile strength is about 40%, the increase rate of elongation at break is about 30%, and the increase rate of the tensile set is about 15%. The increase in the thickness of the compatibility layer can provide a microscopic explanation for the improvement of mechanical properties.

## Figures and Tables

**Table 1:** TPSiV dynamic vulcanization experimental formula (unit: phr) ^(a)^.

Entry	MVSR	SEBS	SBS	KH-907	Platinum Catalyst
1	40	30	30	1	0.5
2	50	25	25	1	0.5
3	60	20	20	1	0.5
4	70	15	15	1	0.5
5	40	30	30	0	0.5
6	50	25	25	0	0.5
7	60	20	20	0	0.5
8	70	15	15	0	0.5

^(a)^ Unless otherwise specified, all samples contain 10 units of white oil and 1 unit of hydrogen-containing silicone oil.

**Table 2:** Infrared characteristic peaks of MVSR and SEBS/SBS.

Abbreviation	Peak Position (cm^−1^)	Corresponding Groups and Vibration Types
MVSR	2965	Stretching vibration of CH_3_ on siloxane
	1260	Stretching vibration of Si(CH_3_)_2_
	1010	Stretching vibration peak of Si-O-Si
SEBS/SBS	2920, 2850	Stretching vibration of CH_3_ group in SEBS/SBS
	760, 700	Characteristic vibration of monosubstituted benzene

**Table 3:** Peak wave numbers of FI-IR diagrams of 100% MVSR, 50% SEBS + 50% SBS and samples 1–8.

Abbreviation	Peak Position (cm^−1^)	Abbreviation	Peak Position (cm^−1^)
MVSR	1010, 1259	50%SEBS + 50%SBS	1261
**Sample No.**	**Peak position (cm^−1^)**	**Sample No.**	**Peak position (cm^−1^)**
1	1018, 1261	5	1016, 1261
2	1016, 1261	6	1014, 1260
3	1014, 1260	7	1014, 1259
4	1014, 1259	8	1012, 1259

**Table 4:** Compatibility layer thickness of samples 1–8.

Sample No.	Thickness of Compatible Layer (μm)	Sample No.	Thickness of Compatible Layer (μm)
1	0.5616	5	0.4143
2	0.4439	6	0.3304
3	0.3518	7	0.2632
4	0.1276	8	0.0963

**Table 5:** Mechanical properties of samples 1–8.

Sample No.	Tensile Strength (MPa)	Break Elongation (%)	Tensile Set Rate (%)
1	9.94	664.74	17.74
2	8.83	583.71	17.89
3	7.17	456.06	24.58
4	4.09	380.01	29.09
5	7.16	516.34	20.82
6	6.52	442.34	21.53
7	5.03	352.80	28.92
8	2.94	296.37	34.21

**Table 6:** The improvement rate of mechanical properties of TPSiV after the addition of silane coupling agent KH-907.

Silicone Rubber Content (phr)	Tensile Strength Improvement Rate (%)	The Rate of Increase in Elongation at Break (%)	Tensile Set Improvement Rate (%)
40	38.83	28.74	14.79
50	35.43	31.96	16.91
60	42.55	29.27	15.01
70	39.12	28.22	14.97

## Data Availability

The data presented in this study are available on request from the corresponding author.
